# Integrative GWAS and Transcriptome Analysis Reveal Genetic Architecture and Candidate Genes for Drought Tolerance During Soybean Germination

**DOI:** 10.3390/plants15193036

**Published:** 2026-10-04

**Authors:** Ziwen Xia, Shanxue Du, Ji Zong, Mingjun Zhang, Qiang Zhang, Fangli Song, Fengqin Yuan, Guiqi Li, Shuang Li, Yongce Cao

**Affiliations:** 1 Shaanxi Key Laboratory of Research and Utilization of Resource Plants on the Loess Plateau, College of Life Sciences, Yan’an University, Yan’an 716000, China; 2 Yan’an Academy of Agricultural Sciences, Yan’an 716000, China; 3 Yan’an Douxing Biotechnology Co., Ltd., Yan’an 716000, China

**Keywords:** soybean, drought tolerance, germination, genome-wide association study, transcriptome, candidate gene

## Abstract

Drought stress severely limits soybean production, and understanding the genetic basis of drought tolerance at the germination stage is important for breeding tolerant varieties. In this study, a panel of 270 soybean accessions was used for phenotypic evaluation and a genome-wide association study (GWAS) of five drought tolerance indices under control and PEG-simulated drought conditions. Using single-locus and multi-locus GWAS models, we detected 109, 115, 121, 86, and 46 significant SNPs, and 14, 7, 9, 10, and 3 QTNs for the five indices, respectively. Combining the results from both models, 97 important QTNs consisting of those controlling multiple traits under the single-locus model and those detected by both models were identified. These QTNs were distributed across 14 genomic intervals, with qDT-4-2 and qDT-9-1 which were associated with all five indices, being the key loci. Transcriptome analysis revealed 2109 genes that were commonly differentially expressed in three tolerant accessions under PEG treatment compared with control conditions. GO and KEGG enrichment analyses showed significant enrichment of pathways related to cell wall metabolism, osmotic adjustment, and antioxidant defense, including amino sugar and nucleotide sugar metabolism, starch and sucrose metabolism, galactose metabolism, and ascorbate and aldarate metabolism. Integration of GWAS and transcriptome data predicted 12 candidate genes within these loci, which may function in drought stress responses through regulating cell wall-modifying enzyme activity, accumulating osmoprotectants, and scavenging reactive oxygen species. Our findings provide useful information for understanding the genetic regulatory network of drought tolerance during soybean germination and offer candidate gene resources for marker-assisted breeding and functional gene discovery.

## 1. Introduction

Drought stress has emerged as one of the most devastating abiotic constraints on global food production under accelerating climate change [1]. Both the frequency and intensity of drought events have increased markedly across many parts of the world, and arid and semi-arid regions now account for a substantial proportion of the globe’s arable land [2]. Extreme-weather disasters such as drought have repeatedly caused substantial crop production losses worldwide [3]. In China, drought-prone areas are widely distributed, and water deficit repeatedly causes significant yield losses in major cropping regions, threatening national food security. Among the various strategies to cope with drought, breeding cultivars with genetically improved drought tolerance is widely regarded as one of the most cost-effective and sustainable approaches [4]. Consequently, dissecting the genetic architecture of drought tolerance and identifying the underlying functional genes are essential prerequisites for effective molecular breeding.

Soybean (*Glycine max* (L.) Merr.) is a globally important legume crop that provides high-quality protein and oil, but its growth and productivity are highly susceptible to water deficit [5]. Drought stress can reduce soybean seed weight per plant by an average of 16% under short-duration drought stress and by more than 40% under long-duration drought conditions [6]. Seed germination is the critical initial stage of the plant life cycle, and inhibited germination under drought stress results in uneven seedling emergence and substantial yield reduction [7]. Therefore, focusing research on this critical developmental window is essential for improving stand establishment and yield stability in drought-prone environments. Polyethylene glycol (PEG-6000) has been widely adopted as an osmoticum to simulate drought stress under controlled conditions, because its high molecular weight prevents cell penetration and it is non-toxic to seeds [8]. The application of 15–20% PEG-6000 has been shown to reliably distinguish soybean genotypes based on germination percentage, radicle length, and seedling vigor, thus providing a robust system for large-scale evaluation of germination stage drought tolerance [9,10].

Drought tolerance at the germination stage of soybean is a complex quantitative trait controlled by multiple genes and regulated by genotype-by-environment interactions [11,12,13]. Both biparental QTL mapping and genome-wide association study (GWAS) have been adopted to dissect the genetic mechanisms underlying soybean drought tolerance during germination. Aleem et al. [11] conducted GWAS with PEG-simulated drought stress and identified many SNP markers significantly associated with germination rate and radicle length. Kwon et al. [12] mapped a major QTL on chromosome 10 that governs slow wilting at the early vegetative stage under drought in a recombinant inbred line population, and this QTL could explain 9.1–11.3% of the phenotypic variation. Zhao and Liu [13] performed association analysis on multiple germination traits under drought and detected drought-tolerant genetic loci on multiple chromosomes. Collectively, these studies demonstrate that while progress has been made in locating genomic regions for germination-stage drought tolerance, the number of major effect and crucial loci remains limited, which severely constrains their direct application in marker-assisted selection and map-based gene cloning [13,14,15].

Seed germination under osmotic stress in plants is primarily regulated through the antagonistic interactions of abscisic acid (ABA) and gibberellins (GA), which govern the dormancy-to-germination transition, as well as through osmotic adjustment and the activation of antioxidant defense systems [16]. Key components of ABA signaling, such as the bZIP transcription factor ABI5, have been demonstrated to function as critical repressors of seed germination under stress conditions [17]. To date, several drought-resistance genes have been functionally characterized in soybean. Among them, transcription factors constitute a major class of regulatory components. For instance, overexpression of the NAC family member *GmNAC19* enhances antioxidant capacity and suppresses reactive oxygen species (ROS) accumulation, which in turn significantly improves germination rate and radicle length under PEG-simulated osmotic stress [18]. Similarly, the bZIP transcription factor *GmbZIP60* reinforces drought tolerance by modulating ABA signaling and promoting proline biosynthesis [19]. However, not all identified regulators act during early development; some, such as *GmNFYB17* and *GmERF113*, appear to function primarily at the seedling or whole plant stage [20,21]. The MYB factor *GmMYB14* offers another example—it enhances drought tolerance by regulating plant architecture through the brassinosteroid pathway—yet its specific role during seed germination remains unexplored [22]. Beyond these individual actions, cross-regulation also plays a key role; the WRKY protein GmWRKY27 interacts with GmMYB174 to repress *GmNAC29* expression, thereby improving overall plant stress tolerance [23]. To identify novel candidate genes without prior functional clues, integrative multi-omics approaches have been increasingly adopted. For instance, a combined metabolomic and transcriptomic study revealed that pathways related to proline, soluble sugars, and flavonoid metabolism are significantly reprogrammed under drought stress, providing a list of candidate genes for further investigation [24]. Another study integrated QTL mapping with transcriptomic data and identified several differentially expressed genes within the QTL intervals that may contribute to drought tolerance during the early growth stage [15]. Despite these advances, the specific molecular regulatory networks that operate during seed imbibition and radicle protrusion under water deficit remain poorly understood, and the number of experimentally validated genes for germination-stage drought tolerance is still extremely small [15,24]. Thus, more candidate genes need to be identified and functionally verified through integrated approaches.

The Loess Plateau region of northern Shaanxi is characterized by erratic spring rainfall, and recurrent drought during the soybean sowing period severely impairs seed germination and seedling establishment, posing a major bottleneck for local soybean production. To address this, a diverse panel of 270 soybean accessions was evaluated for germination-stage drought tolerance using 15% PEG-6000 to simulate osmotic stress, and subsequently GWAS and transcriptomic profiling were performed on the superior accessions. The objectives of this study were to dissect the genetic architecture underlying drought tolerance at the germination stage, identify key molecular markers and candidate genes along with their associated regulatory pathways, and thereby provide novel genetic resources and a theoretical basis for the molecular breeding of drought-tolerant soybean cultivars in northern Shaanxi.

## 2. Results

### 2.1. Phenotypic Evaluation and Analysis of Drought Tolerance-Related Traits

**Figure 1:**
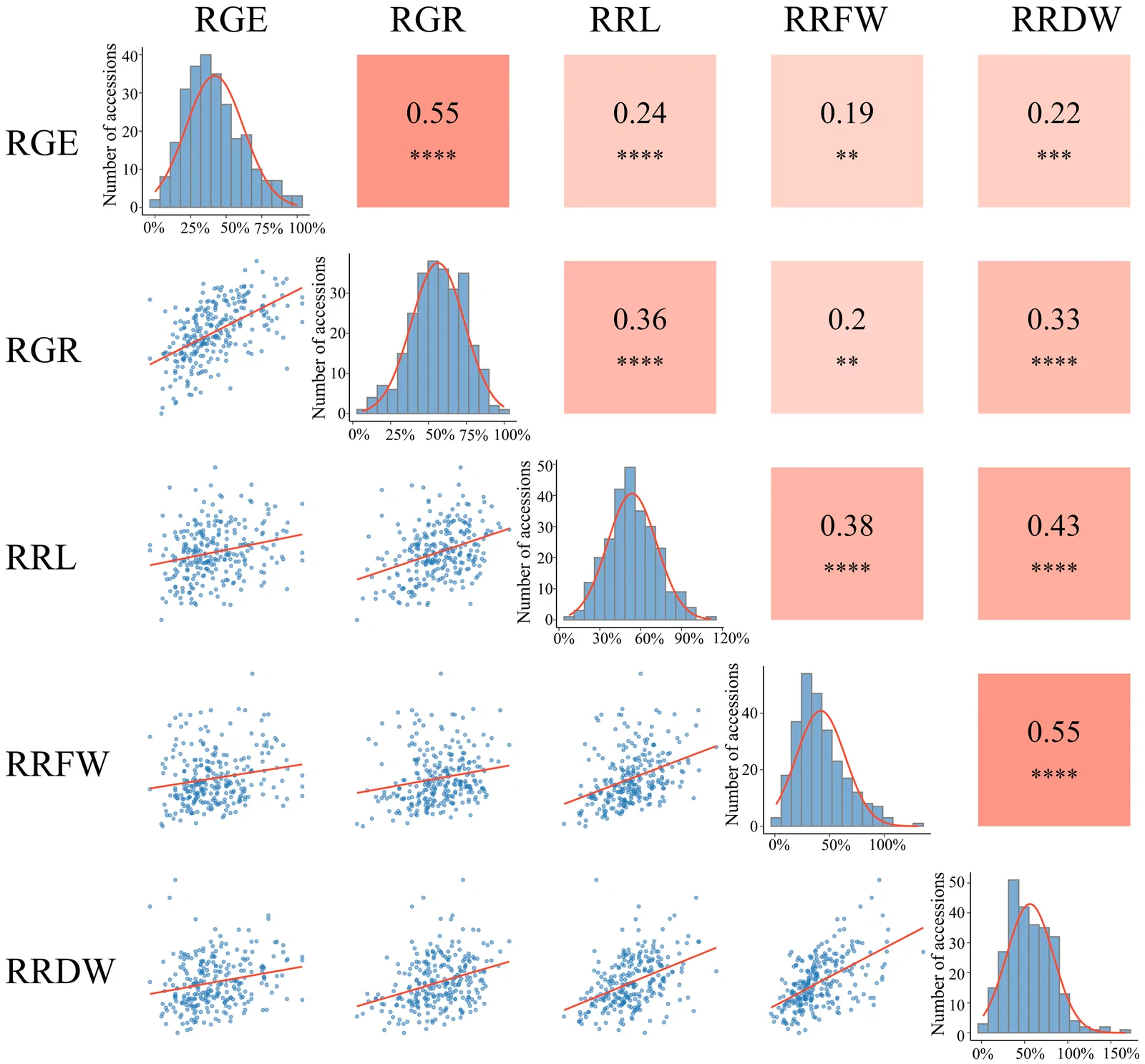
Phenotypic distribution and correlation analysis of five drought tolerance indices in soybean accessions. The diagonal panels show frequency distribution histograms for each drought tolerance index; the lower triangular panels show linear regression lines for pairwise relationships between traits; the upper triangular panels show Pearson correlation coefficients between traits, with darker background colors indicating larger values. ** *p* < 0.01, *** *p* < 0.001, **** *p* < 0.0001. RGE, relative germination energy; RGR, relative germination rate; RRL, relative radicle length; RRFW, relative radicle fresh weight; RRDW, relative radicle dry weight.

Five drought-related traits at the germination stage of 270 soybean accessions were evaluated under CK and 15% PEG-6000 treatment conditions (Figure 1 and Table 1). Under the CK condition, the mean values ± SD of GE, GR, RL, RFW and RDW were 49.08 ± 17.83%, 58.82 ± 18.50%, 5.60 ± 2.76 cm, 124.39 ± 61.40 mg, and 11.84 ± 7.05 mg, respectively, with coefficients of variation (CV) ranging from 31.45% to 59.55%. Under the PEG treatment, the mean values ± SD of the above traits were 19.91 ± 12.87%, 33.55 ± 17.58%, 2.59 ± 1.35 cm, 39.83 ± 18.97 mg, and 6.12 ± 2.98 mg, respectively, with CV values ranging from 47.62% to 64.63%. Compared with the CK group, the mean values of all traits under PEG treatment decreased by 59.43%, 42.96%, 53.74%, 67.98% and 48.31%, respectively. These results indicate that PEG stress significantly inhibits soybean seed germination, reflecting the impact of drought stress on seed germination.

To exclude the influence of intrinsic germination ability on drought tolerance evaluation, the relative values of each trait under the two treatments (i.e., drought tolerance indices, RGE, RGR, RRL, RRFW and RRDW) were further used for subsequent analyses, and their distributions are shown in Table 1 and Figure 1. The mean values ± SD of RGE, RGR, RRL, RRFW and RRDW were 41.98 ± 20.30%, 55.76 ± 17.95%, 53.69 ± 17.89%, 42.15 ± 22.58% and 57.01 ± 28.58%, respectively, with CV values ranging from 32.20% to 53.57%. Shapiro–Wilk tests showed that RGR and RRL were normally distributed (*p* > 0.05), whereas RGE, RRFW, and RRDW did not pass the normality test (*p* < 0.05). Nevertheless, all five drought tolerance indices exhibited continuous distributions and showed abundant genetic variation, indicating that the population used in this study is suitable for reflecting the genetic basis of drought tolerance in soybean. Thirteen accessions ranked in the top one-third for all five indices and were therefore considered drought-tolerant.

To explore the correlations among the five drought tolerance indices, Pearson’s correlation analysis was performed on the RGE, RGR, RRL, RRFW and RRDW values (Figure 1). The results showed that all five indices were significantly positively correlated with each other (*p* < 0.01), with correlation coefficients ranging from 0.19 to 0.55, indicating moderate correlations. These results suggest that while these drought tolerance traits share certain commonalities, they also exhibit distinct characteristics, and thus can collectively reflect the drought tolerance of soybean at the germination stage from different aspects.

### 2.2. Detection of QTNs Using Single-Locus and Multi-Locus GWAS Models

**Figure 2:**
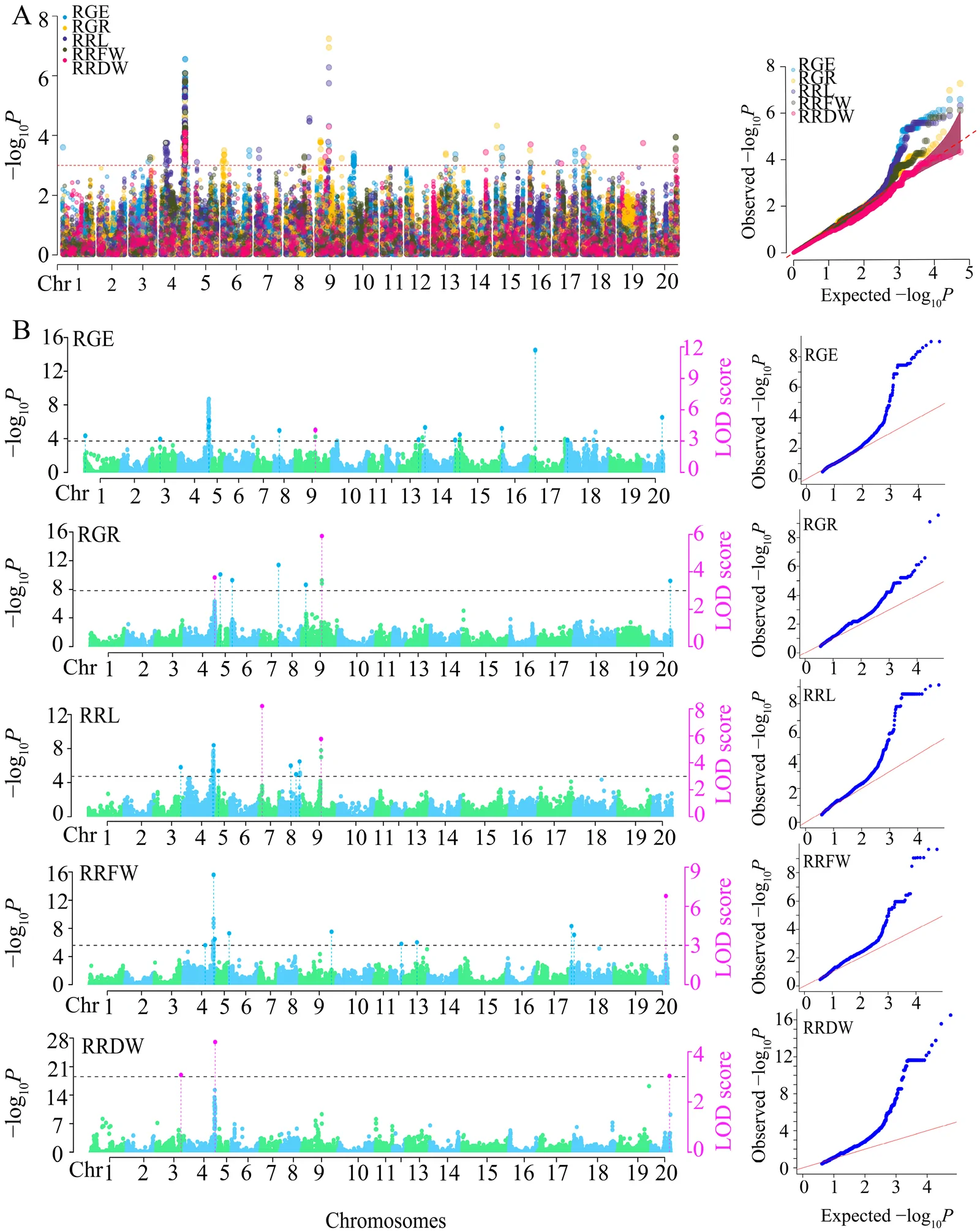
Genome-wide association study (GWAS) results for five drought-tolerance indices by single-locus GWAS and multi-locus GWAS models. (**A**) Results of single-locus GWAS model. (**B**) Results of multi-locus GWAS models.

**Figure 3:**
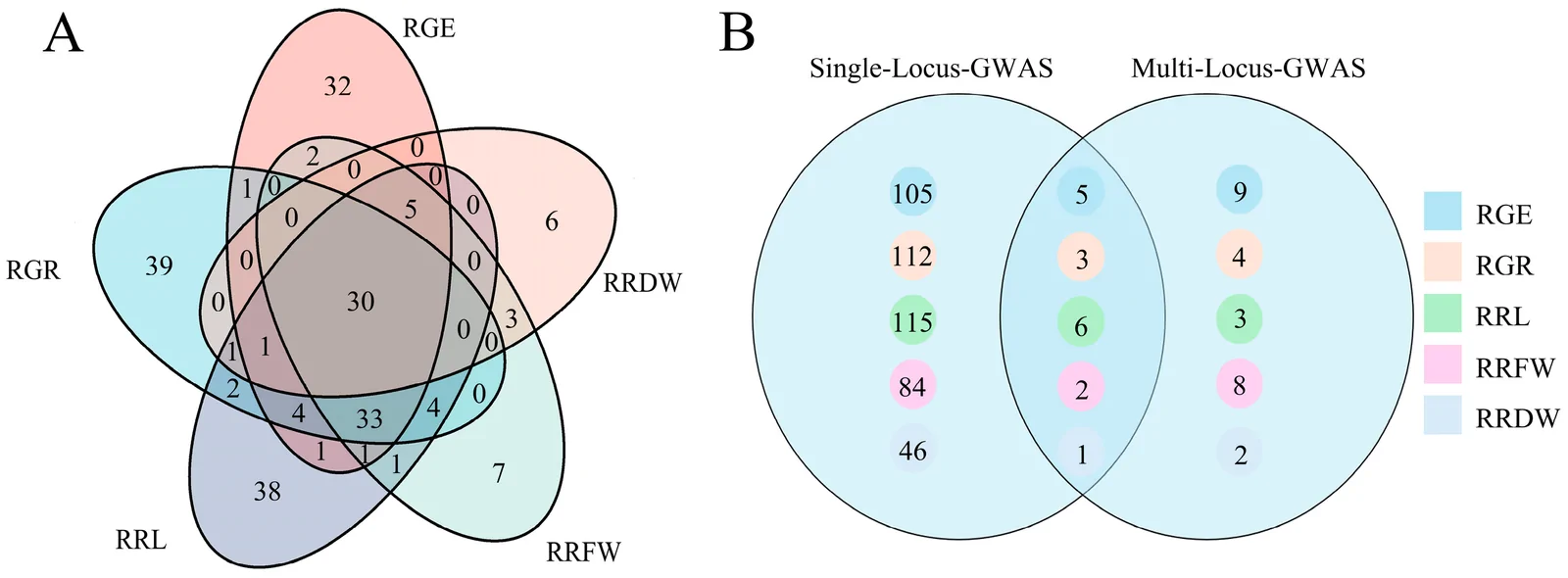
Summary of GWAS results for five drought tolerance indices. (**A**) QTNs detected for the five indices using the single-locus model. (**B**) Comparison of results between the single-locus GWAS model and the multi-locus GWAS model. Numbers indicate the number of detected QTNs.

In this study, single-locus and multi-locus GWAS models were used to perform marker-trait association analyses for five drought-tolerance indices at the germination stage to detect QTNs (Figure 2 and Figure 3, Tables S1 and S2).

Under the single-locus GWAS model (MLM), with a threshold of −log_10_(*P*) ≥ 3, 109, 115, 121, 86, and 46 SNPs were detected to be significantly associated with RGE, RGR, RRL, RRFW, and RRDW, respectively (Figure 2 and Table S1). After removing redundant SNPs, a total of 210 drought tolerance QTNs were identified based on the single-locus model. The −log_10_(*P*) values for individual SNPs ranged from 3.00 to 7.24, and the allelic effect values ranged from −15.06% to 10.94%. Comparing the genome-wide association results across the five drought-tolerance indices showed that 30, 39, 10, and 10 significant SNPs simultaneously affected 5, 4, 3, and 2 traits, respectively, and these were considered important SNPs controlling multiple drought-tolerance indices (Figure 3).

To further validate and supplement the results from the single-locus GWAS model, three multi-locus GWAS models were applied. For the five drought-tolerance indices (RGE, RGR, RRL, RRFW, and RRDW), 14, 7, 9, 10, and 3 QTNs were detected, respectively (Figure 2 and Table S2). The effect values for individual QTNs ranged from −11.27% to 8.00%, the logarithm of odds (LOD) scores ranged from 3.02 to 11.70, and the phenotypic variation explained (PVE) ranged from 0.03% to 10.02%. Comparison of the results from the two types of GWAS models (single-locus and multi-locus) showed that 5 (RGE), 3 (RGR), 6 (RRL), 2 (RRFW), and 1 (RRDW) QTNs were detected by both strategies, and these were considered robust and important drought-tolerance QTNs (Figure 3).

Combining the results from both single-locus and multi-locus GWAS models, a total of 97 important QTNs were identified. Based on LD analysis of markers in the upstream and downstream regions of significant QTNs, the genomic intervals containing these QTNs were defined. The results showed that these 97 QTNs were distributed across 14 QTLs. Therefore, these 14 QTLs were considered key loci for drought tolerance in soybean at the germination stage (Table 2 and Figure S1).

**Figure 4:**
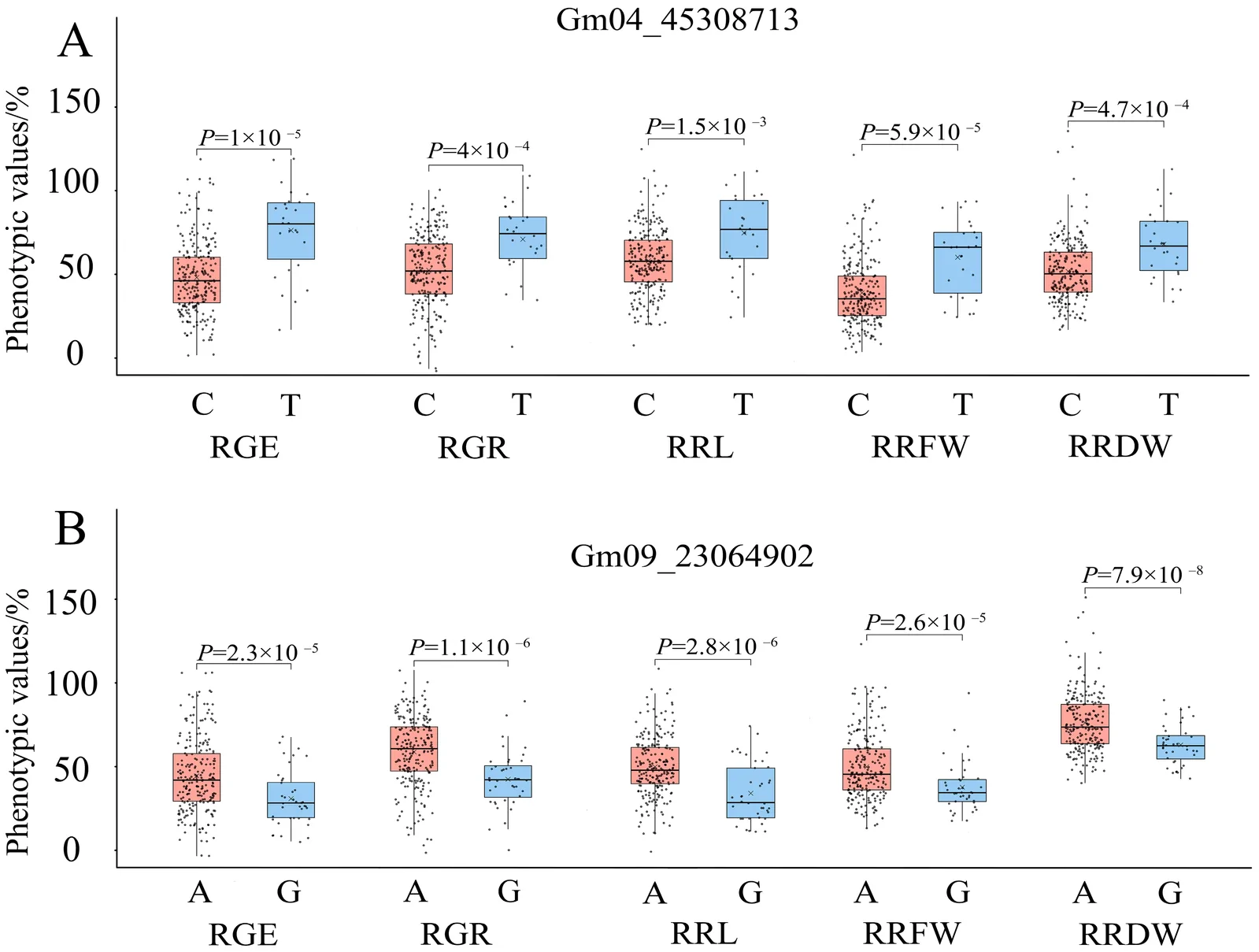
Distributions of five drought-tolerance indices among individuals with different alleles of the peak QTNs within the two most important QTLs. (**A**) Gm04_45308713 (qDT-4-2). (**B**) Gm09_23064902 (qDT-9-1).

Among them, two QTLs (qDT-4-2 and qDT-9-1) simultaneously controlled all five drought-tolerance indices and were the most important loci in this population. To validate the effects of these loci and screen for favorable allelic variants, the most significantly associated SNP markers (Gm04_45308713 and Gm09_23064902) within these two loci were selected for analysis of the phenotypic distribution of drought-tolerance indices under different alleles (Figure 4). The results showed that accessions carrying the T allele of Gm04_45308713 (*n* = 26) had mean phenotypic values for RGE, RGR, RRL, RRDW, and RRFW of 63.66%, 69.25%, 67.12%, 63.35%, and 79.14%, respectively, all of which were significantly higher than those of the C-allele group (*n* = 244, 39.52%, 54.33%, 52.26%, 39.88%, and 54.64%, respectively; all *p* < 0.01, Wilcoxon rank-sum test). For Gm09_23064902, accessions with the A allele (*n* = 230) exhibited mean phenotypic values of 43.86%, 57.84%, 55.92%, 44.28%, and 60.17% for RGE, RGR, RRL, RRDW, and RRFW, respectively, which were higher than those of the G-allele group (*n* = 40) by 12.78, 14.03, 15.07, 14.68, and 21.79 percentage points, respectively (all *p* < 0.001, Wilcoxon rank-sum test). The significant genetic effect divergence between different alleles at these two loci confirmed that they are key loci for drought-tolerance-related traits in soybean.

### 2.3. Identification and GO/KEGG Enrichment Analysis of Differentially Expressed Genes

Based on the phenotypes of five drought tolerance indices, three drought-tolerant accessions, DT1, DT2, and DT3, were selected for transcriptome analysis in this study. The RGE, RGR, RRL, RRFW, and RRDW of these accessions ranged from 61.75% to 89.71%, 84.19% to 89.18%, 62.77% to 85.20%, 50.30% to 76.21%, and 68.83% to 99.39%, respectively, and all indices ranked in the top one-third among the tested soybean accessions. A total of 18 samples were then subjected to transcriptome sequencing and stringent quality control. In total, 134.22 Gb of clean data were generated, with 6.34–8.79 Gb of clean bases per sample. The sequencing error rate was 0.02%, and Q20 and Q30 were higher than 98.87% and 97.77%, respectively. The GC content ranged from 43.41% to 43.85% (Table S3). To further evaluate sample reproducibility and inter-group differences, correlation analysis and principal component analysis (PCA) were performed on the transcriptome data of the 18 samples (Figure S2). Correlation analysis showed that the Pearson correlation coefficients among the three biological replicates within the same genotype and treatment were consistently high, indicating good sample reproducibility. PCA showed that biological replicates within the same genotype and treatment clustered tightly, whereas different genotypes and treatment groups were clearly separated, indicating that genotype and treatment were the main factors driving transcriptome variation.

**Figure 5:**
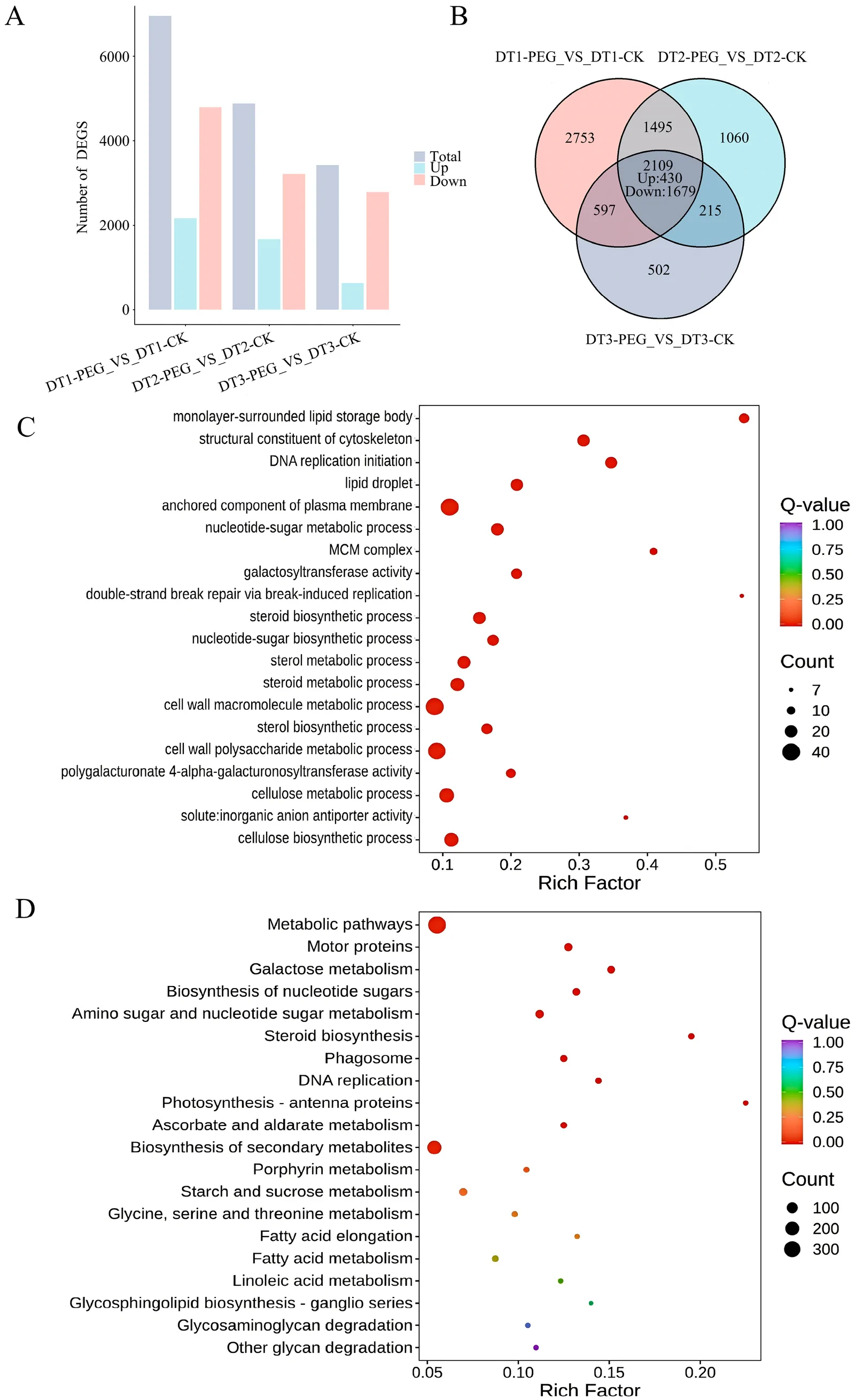
Identification and enrichment analysis of differentially expressed genes (DEGs). (**A**) DEGs identified in the PEG vs. CK comparison groups for three drought-tolerant accessions. (**B**) Number of shared DEGs among the three comparison groups. (**C**) GO enrichment results of the shared DEGs. (**D**) KEGG enrichment results of the shared DEGs.

Using the thresholds of |log_2_FC| ≥ 1 and *P*_adj_ < 0.05, by comparing the PEG-treated group with the CK control, a total of 6854 DEGs (2163 upregulated and 4791 downregulated) were identified for the DT1 accession; 4879 DEGs (1666 upregulated and 3213 downregulated) were obtained for DT2; and 3423 DEGs (635 upregulated and 2788 downregulated) were detected for DT3. Comparative analysis of the DEGs among the three accessions revealed that 2109 genes were commonly differentially expressed across all three accessions, of which 430 were upregulated and 1679 were downregulated (Figure 5 and Table S4). These results indicate that a large number of genes are repressed under PEG-simulated osmotic stress during soybean seed germination, which is a typical stress-response characteristic of plants under drought conditions. To further elucidate the molecular mechanisms underlying drought tolerance in soybean at the germination stage, GO functional annotation and KEGG pathway enrichment analyses were performed using these 2109 common DEGs (Figure 5, Tables S5 and S6).

GO functional enrichment analysis revealed that the differentially expressed genes (DEGs) were significantly enriched in functional categories closely related to drought tolerance, including cell wall polysaccharide metabolism, sterol biosynthesis, and water transport (Figure 5C and Table S5). In the biological process category, terms such as cell wall macromolecule metabolic process (GO:0044036), cell wall polysaccharide metabolic process (GO:0010383), and plant-type cell wall biogenesis (GO:0009832) were highly enriched, indicating that soybean seeds reinforce physical barriers by remodeling cell wall composition under drought stress. Meanwhile, sterol biosynthetic process (GO:0006694), sterol metabolic process (GO:0016125), and lipid droplet-related terms (GO:0012511, GO:0005811) were significantly enriched, suggesting that maintenance of membrane lipid homeostasis and lipid mobilization play important roles in the drought response. At the molecular function level, enrichment of terms including cellulose synthase (GO:0016759), galactosyltransferase (GO:0008378), UDP-glucose 6-dehydrogenase (GO:0003979), and aquaporin (GO:0015250) further supported the coordinated regulation of cell wall synthesis, osmotic adjustment, and water transport. Moreover, the significant enrichment of the term “response to desiccation” (GO:0009269) itself provided direct evidence linking these functional changes to drought tolerance. Further GO enrichment analysis was performed separately on the commonly upregulated (430) and downregulated (1679) DEGs to dissect their biological functions associated with the response of germinating soybean seeds to PEG-simulated drought stress (Tables S7 and S8). The commonly downregulated DEGs were significantly enriched in processes such as cell wall polysaccharide metabolism, steroid/sterol biosynthesis, cytoskeleton constitution, and DNA replication, with representative terms including cell wall macromolecule metabolic process (GO:0044036), cellulose metabolic process (GO:0030245), steroid biosynthetic process (GO:0006694), and MCM complex (GO:0042555). In contrast, the commonly upregulated DEGs were mainly enriched in lipid storage, seed maturation, galactose metabolism, and transmembrane transport, with representative terms including lipid droplet (GO:0005811), monolayer-surrounded lipid storage body (GO:0012511), seed maturation (GO:0010431), galactose metabolic process (GO:0006012), and passive transmembrane transporter activity (GO:0022803). These results indicate that the functional enrichment patterns of commonly upregulated and downregulated DEGs were markedly different (Figure S3).

KEGG pathway enrichment analysis showed that the DEGs were significantly (*p* < 0.05) enriched in multiple drought-responsive metabolic pathways (Figure 5D and Table S6). In carbohydrate metabolism, galactose metabolism (ko00052), amino sugar and nucleotide sugar metabolism (ko00520), and starch and sucrose metabolism (ko00500) were all significantly enriched, suggesting that seeds carry out osmotic adjustment by breaking down storage polysaccharides to generate soluble sugars. In lipid metabolism, steroid biosynthesis (ko00100) was significantly enriched, and fatty acid metabolism (ko01212) as well as cutin, suberine and wax biosynthesis (ko00073) were also enriched, reflecting the important role of membrane stability regulation in drought tolerance. In addition, ascorbate metabolism (ko00053) was significantly enriched, suggesting that antioxidant defense was activated and involved in the response to drought stress. However, when KEGG enrichment analysis was performed separately on the commonly upregulated and downregulated DEGs, no statistically significant pathways were identified.

Combining the GO and KEGG enrichment results, the two approaches corroborated and complemented each other. For instance, the enrichment of cell wall-related and sterol-lipid-related GO terms corresponded well with the activation of starch/nucleotide sugar metabolism pathways and steroid/fatty acid metabolism pathways in KEGG, respectively, together reflecting the coordinated responses of osmotic adjustment, cell wall remodeling, and membrane lipid homeostasis. These findings preliminarily reveal the molecular regulatory pathways through which soybean resists drought stress during germination.

### 2.4. Candidate Gene Mining by Integration of GWAS and RNA-Seq

**Figure 6:**
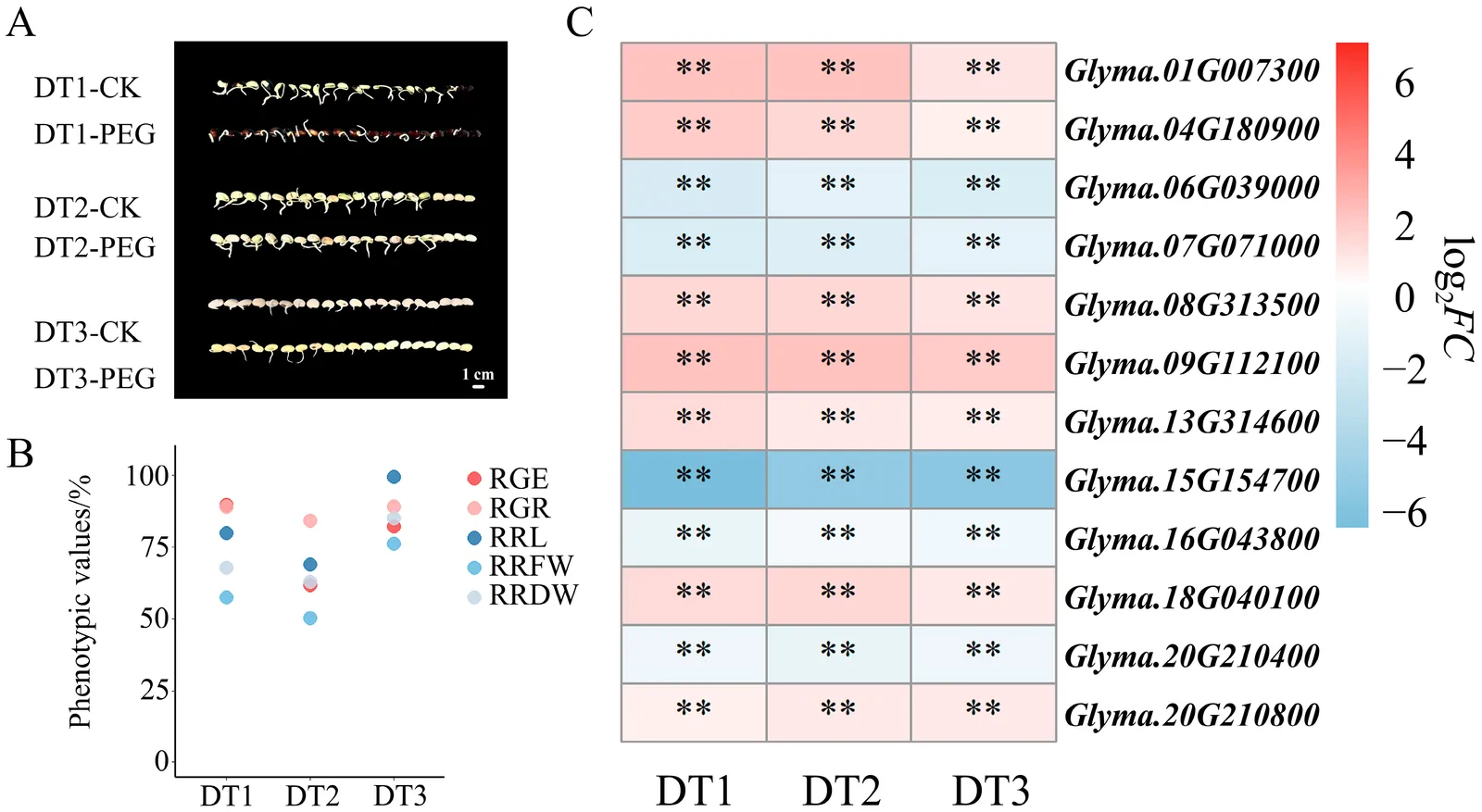
Phenotypes and candidate gene expression profiles of three drought-tolerant soybean accessions. (**A**) Germination performance of DT1, DT2, and DT3 under normal conditions (CK) and PEG-simulated drought treatment (PEG). (**B**) Phenotypic values of five drought tolerance indices (RGE, RGR, RRL, RRFW, and RRDW) for the three accessions. (**C**) Differential expression of 12 candidate genes in DT1, DT2, and DT3 under different treatments. ** *p* < 0.01.

Candidate genes associated with drought tolerance at the soybean germination stage were mined within 14 key loci detected by GWAS. However, after integration with transcriptome data from drought-tolerant germplasms under different treatments, only 42 genes showed stable differential expression with |log_2_FC| ≥ 0.5 and *P*_adj_ < 0.05 across all three soybean accessions (Table S9). These genes were therefore retained as candidates for further analysis. Through the integration of gene functional annotation, GO/KEGG enrichment analysis, and previously published literature, 12 candidate genes potentially playing critical roles in regulating drought tolerance at the soybean germination stage were ultimately predicted (Figure 6 and Table 3). Among them, *Glyma.09G112100*, *Glyma.04G180900*, *Glyma.16G043800*, and *Glyma.18G040100* were found to encode a late embryogenesis abundant (LEA) protein, trehalose-6-phosphate synthase, an aquaporin-like superfamily protein, and dehydroascorbate reductase, respectively, and were suggested to function in osmotic regulation, water transport and reactive oxygen species scavenging. The remaining candidate genes may be involved in hormone signal transduction or calcium signaling cascades. Overall, these genes may contribute to the regulation of drought tolerance during soybean germination through multiple pathways and thus represent promising candidate genes.

## 3. Discussion

Drought stress is a major ecological constraint that severely impairs plant growth and development, leading to substantial losses in global agricultural productivity [3]. Among legume crops, soybean is particularly sensitive to water availability and requires adequate moisture throughout its entire growth cycle [29,30]. Seed germination is the initial and most vulnerable stage of the life cycle, and drought stress during this stage can cause uneven emergence and poor stand establishment in soybean and consequently pose a serious threat to final yield [29,31]. This challenge is especially pronounced in the Loess Plateau region of northern Shaanxi, where soybean sowing (from late April to early May) frequently coincides with spring drought. Therefore, without increasing irrigation inputs, breeding soybean varieties with enhanced drought tolerance at the germination stage represents a feasible strategy for stabilizing local production. The method of using PEG-6000 to simulate drought stress has been widely validated as an effective means to mimic water deficit during seed germination [9,10]. Our experimental results also showed that PEG treatment significantly inhibited soybean seed germination, consistent with previous reports that osmotic stress restricts radicle protrusion and seedling growth [11,14], confirming the suitability of this system for large-scale phenotypic evaluation. However, during seed stress tolerance evaluation, some accessions exhibited low germination rates, possibly due to differences in seed maturity and dormancy characteristics [14,32]. Similar results were observed in this study. Therefore, additional phenotypic evaluation replicates were included to reduce the influence of random error. Overall, the drought-tolerance indices of the 270 soybean accessions examined all exhibited continuous approximately normal distributions with ample phenotypic variation, indicating that this panel possesses sufficient genetic diversity to support subsequent efforts in drought-tolerant germplasm screening, genetic architecture dissection, and candidate gene discovery.

Soybean drought tolerance at the germination stage is a complex quantitative trait governed by multiple genes, and its genetic basis has been extensively dissected [11,12,13,14,15,33]. Despite the identification of numerous QTLs/QTNs, their utility in marker-assisted selection and gene functional studies is largely constrained by phenotypic evaluation and detection approaches [13,14,15,33]. Consequently, consistently detected major-effect loci remain to be identified. In the present study, based on five drought-tolerance indicators and using both single-locus and multi-locus GWAS models, we identified 97 important QTNs distributed across 14 genomic intervals (QTLs), which exhibited consistent stability across traits or models. Comparative analysis with previous reports revealed that several loci were adjacent to or overlapped with previously identified drought-related QTLs/QTNs. Specifically, qDT-4-1, qDT-13-1, qRGE-15-1, and qDT-16-1 were in close proximity to qGR4 (Gm04-8805064) identified by Aleem et al. [11], qRGP-13-1 reported by Kong et al. [14], QTN-Gm15_11950665 reported by Zhao et al. [13], and qWSL16 (Gm16-3107434) detected by Aleem et al. [11], respectively. Furthermore, qDT-4-1 was also located near the drought-related QTL Canopy wilt 2-2 [25] from other growth stages, while qRRL-5-1 completely coincided with the seedling-stage QTLs Canopy wilt 2-3 [25] and Drought tolerance 6-2 [27]. These findings further corroborate the functional relevance of these genomic regions in soybean drought tolerance regulation.

Notably, qDT-4-2, qRGE-18-1, and qDT-20-1 overlapped with root-development QTLs—namely, Root morphology 1-3 [26], Root density, lateral 1-1 [28], and Root morphology 1-5 [26]—implying that these loci may contribute to drought tolerance through modulating root architecture. In addition, we identified previously unreported drought-tolerance-related QTLs, such as qRGE-1-1 and qDT-9-1, along with others, thereby further broadening the genetic basis of drought tolerance during soybean germination. Taken together, the key loci detected in this study provide promising targets for molecular breeding and gene cloning for drought tolerance in soybean.

Transcriptome sequencing has become a routine strategy for elucidating the molecular mechanisms underlying plant abiotic stress tolerance [33,34]. In this study, transcriptome data from three drought-tolerant soybean accessions under PEG-simulated drought stress combined with GO functional annotation and KEGG pathway enrichment analysis, were analyzed to explore the molecular regulatory mechanisms of drought stress response during soybean seed germination. The results showed that carbohydrate metabolism and osmoregulation pathways (ko00500, ko00052, ko00260) were significantly enriched. Activation of carbohydrate metabolism pathways facilitates the breakdown of storage polysaccharides to generate soluble sugars and compatible solutes, thereby reducing cellular osmotic potential and maintaining water uptake. This is consistent with the well-established strategy that carbohydrate metabolism confers osmotic adjustment under osmotic stress [35,36]. Cell wall synthesis and epidermal wax deposition are important mechanisms for enhancing plant drought and salt tolerance [37,38,39]. In this study, pathways involved in cell wall synthesis and cutin, suberine and wax biosynthesis (ko00520 and ko00073) were significantly enriched, suggesting that soybean seeds may reinforce physical barriers and reduce water loss through coordinated regulation of cell wall remodeling and cuticular wax deposition, thereby enhancing drought tolerance during germination. Antioxidant defense and membrane system stability maintenance are critical mechanisms in plant drought stress responses [40,41]. The enrichment of ascorbate and aldarate metabolism (ko00053) in this study may function in alleviating oxidative damage by scavenging reactive oxygen species, while steroid biosynthesis (ko00100) may play a role in drought responses, potentially by influencing membrane properties. Furthermore, separate enrichment analysis of upregulated and downregulated DEGs indicated that drought-tolerant soybean accessions may actively suppress growth-related processes such as cell division and DNA replication under drought stress, thereby reducing water and energy consumption. In contrast, upregulated DEGs were associated with lipid storage, seed maturation, and transmembrane transport, which may help maintain cellular homeostasis and stress defense. Collectively, the significant enrichment of multiple biological pathways associated with plant drought stress responses in this study provides a comprehensive view of the molecular regulatory mechanisms underlying drought tolerance during soybean seed germination.

Drought tolerance in soybean is a quantitative trait controlled by multiple genes [33,42]. In recent years, although some genes involved in drought tolerance regulation in soybean have been identified, most were discovered at the seedling or mature stages. In this study, we integrated GWAS and transcriptome sequencing results and predicted a total of 12 candidate genes associated with germination-stage drought tolerance. Among these candidates, *Glyma.09G112100* encodes a Late Embryogenesis Abundant (LEA) protein. LEA proteins act as classic molecular protectants by preventing protein aggregation during seed desiccation and rehydration. As seeds transition from quiescence to germination, they gradually lose desiccation tolerance, and LEA proteins play a critical role in defending against low water potential stress during imbibition [43,44,45]. Therefore, this gene represents an important candidate for drought tolerance at soybean germination. Trehalose-6-phosphate (T6P) functions both as an osmolyte and as a key metabolic signal linking sugar availability to growth. Under drought conditions, TPS activity supports osmotic adjustment and protects cellular membranes during dehydration [46]. *Glyma.04G180900*, located within the qDT-4-2 locus, encodes trehalose-6-phosphate synthase (TPS), implying its possible involvement in drought tolerance regulation at this locus. ABA signaling plays a central role in drought responses during seed germination, and PP2C genes function as key regulators in ABA-mediated drought signaling [46,47,48]. *Glyma.06G039000* encodes PP2C family proteins and can therefore be considered a candidate gene. Aquaporins, as molecular channels for transmembrane water transport, directly determine the water acquisition and retention capacity of cells and tissues, and play important roles in crop drought resistance [49]. *Glyma.16G043800* encodes an SIP-subclass aquaporin, indicating its potential involvement in water transport during drought tolerance at soybean germination. In *Arabidopsis*, *JMJ27*-mediated histone H3K9 demethylation positively regulates drought tolerance by epigenetically modulating the expression of drought stress-responsive genes [50]. *Glyma.08G313500*, located within qRRL-8-1, encodes a jmjC domain-containing protein and may participate in the regulation of drought tolerance during soybean germination through the same pathway. NAC transcription factors are plant-specific master regulators of stress responses and are deeply involved in the ABA-dependent drought response network [51,52]. *Glyma.13G314600* encodes a NAC domain-containing protein and may participate in drought tolerance regulation during germination by coordinating the expression of genes related to osmotic protection, ROS scavenging, and ABA signaling. *MYC2*-type bHLH transcription factors are core hubs for crosstalk between jasmonic acid and ABA signaling, and are involved in stomatal regulation, osmotic responses, and stress-related gene expression under drought stress [53,54]. *Glyma.07G071000* encodes a *MYC2*-type bHLH transcription factor and can therefore be considered a candidate gene. *Glyma.20G210800* encodes a CBL-interacting protein kinase 4 (CIPK4), belonging to the CIPK family, most members of which have been shown to be drought-inducible in soybean [55]. Family members *GmCIPK2* and *GmCIPK29* have been confirmed by transgenic experiments to positively regulate drought tolerance in soybean [56,57], and overexpression of the orthologous *MsCIPK4* in alfalfa significantly enhanced seed germination under drought stress [58], suggesting that this gene has a function in germination-stage drought tolerance regulation.

Plant drought tolerance involves the coordinated action of multiple pathways, including hormone signal transduction, osmotic regulation, and reactive oxygen species scavenging [42,59]. *Glyma.01G007300* participate in carbohydrate metabolism and sugar signaling, potentially contributing to osmotic adjustment under drought. *Glyma.20G210400* was involved in auxin responses. *Glyma.15G154700* functions in osmotic, salt, and water-deficit stress responses. *Glyma.18G040100* encodes dehydroascorbate reductase (DHAR), which may act in ROS scavenging. These genes are therefore considered candidate genes. This study conducted transcriptome analysis of the commonly differentially expressed genes (DEGs) from three drought-tolerant accessions under PEG versus CK conditions; however, the analysis alone could not distinguish general PEG-responsive genes from drought-tolerance-specific genes. To identify drought-tolerance-related genes, only DEGs located within key drought-tolerance QTLs were considered as potential candidate genes, yet their functions still require further experimental validation.

## 4. Materials and Methods

### 4.1. Plant Materials

In this study, the association panel previously constructed in our laboratory was used [60,61]. This panel comprises 281 soybean accessions, predominantly collected from Northwest China, North China, and the Huang-Huai-Hai region. All accessions were grown at the experimental station of the Yan’an Institute of Agricultural Sciences, Shaanxi Province (36.72° N, 109.40° E), from May to October 2025. However, due to insufficient seed quantity or substandard seed quality in some accessions, a final set of 270 accessions was utilized for drought tolerance identification at the seed germination stage.

### 4.2. Drought Stress Treatment and Phenotypic Evaluation

Twenty intact seeds of uniform size and free from mechanical damage were selected from each accession. The seeds were surface-sterilized with 75% ethanol for 45 s and rinsed three times with sterile distilled water. Subsequently, the seeds were evenly arranged on double-layer filter paper in Petri dishes of 12 cm diameter.

Based on preliminary PEG-6000 concentration gradient experiments and previously reported methods, 20 mL of 15% (*w*/*v*) PEG-6000 solution was added to each Petri dish in the drought treatment group, while the control group received an equal volume of distilled water [14,62]. All Petri dishes were incubated in the dark at 25 ± 1 °C for 6 days, with three biological replicates per treatment [62]. During the incubation period, 2 mL of the corresponding solution was replenished daily.

Germination was recorded when the radicle length exceeded half of the seed length. The number of germinated seeds was counted every 24 h for 6 consecutive days. On the 6th day, five seedlings with uniform growth vigor were selected from each accession. Radicle length (RL) was measured with a ruler and averaged; the total fresh weight of the five radicles with cotyledons removed was weighed using an electronic balance to calculate the average radicle fresh weight (RFW). Subsequently, the radicle samples were heated at 105 °C for 20 min to inactivate enzymes and then dried at 80 °C for 24 h to constant weight, after which the radicle dry weight (RDW) was recorded. Radicle fresh weight and dry weight were measured using a Mettler Toledo XS105DUE electronic analytical balance with a readability of 0.01 mg. Based on the daily germination counts, germination energy and germination rate were calculated using the following formulas:

Germination energy (GE) = (Number of germinated seeds on the 3rd day/Total tested seeds) × 100%, reflecting germination speed and uniformity. Germination rate (GR) = (Number of germinated seeds on the 6th day/Total tested seeds) × 100%, representing final germination capacity and seed vigor. For accessions with a germination rate below 30% under control conditions, three additional paired replicates, each containing 60 seeds, were included to improve the reliability of phenotypic evaluation and to reduce the influence of random error on drought tolerance evaluation.

The relative value of each trait under stress versus control was used as the drought tolerance index to evaluate the drought tolerance of different accessions. The formula is: Relative trait value = (Trait value under stress/Trait value under control) × 100%. Accordingly, the relative germination energy (RGE), relative germination rate (RGR), relative radicle length (RRL), relative radicle fresh weight (RRFW), and relative radicle dry weight (RRDW) were calculated. For each index, all accessions were divided into three categories based on their phenotypic values: the top one-third as drought-tolerant, the middle one-third as moderate, and the lowest one-third as sensitive. Only accessions ranking in the top one-third for all five indices were selected as drought-tolerant accessions.

### 4.3. GWAS and Key Loci Identification

All accessions used in this study had been previously sequenced and subjected to SNP marker development in our earlier work [60,61]. Briefly, 281 soybean accessions were initially genotyped by reduced-representation genome sequencing (RAD-seq). After removing markers with minor allele frequency (MAF) < 5% and markers with missing and heterozygous allele rates > 30%, a total of 56,608 SNP markers were obtained using Wm82.a2.v1 as the reference genome. In the present study, 270 accessions from the aforementioned population were retained. Ultimately, a total of 56 570 SNP markers with minor allele frequency (MAF) > 0.05 distributed across the whole genome were obtained from the 270 accessions for subsequent analyses.

To dissect the genetic basis of drought tolerance at the germination stage in soybean, two strategies were employed for association analysis between traits and SNPs. The first strategy utilized a single-locus GWAS model, implemented via the mixed linear model (MLM, PCA + K) in the R-GAPIT software package (version 3.5.0) [63]. Based on the quantile–quantile (Q-Q) plots, a threshold of −log_10_(*P*) ≥ 3 was used to screen significantly associated SNP markers [64,65]. This approach was primarily intended to identify as many trait-related SNPs as possible and to evaluate their detection stability across different traits. The second strategy adopted multi-locus GWAS models, including FASTmrMLM, pLARmEB, and ISIS EM-BLASSO, with a LOD (logarithm of odds) score > 3.0 as the threshold for determining significant QTNs [66]. These analyses were performed using the R-mrMLM package [67], and served mainly to verify and complement the results obtained from the single-locus model.

In the single-locus GWAS analysis, SNPs that were simultaneously and significantly associated with multiple traits, or that were detected by the single-locus model and further validated by multi-locus models, were defined as important quantitative trait nucleotides (QTNs) controlling drought tolerance at the soybean germination stage. Subsequently, SNP markers upstream and downstream of the important QTN were selected for linkage disequilibrium (LD) analysis and visualization using Haploview 4.2 [68]. The LD block (genomic region) containing the important QTN was the key locus (Quantitative Trait Locus, QTL). Following previously established QTL naming conventions with minor modifications, a QTL affecting a single trait was named as “q + trait abbreviation + chromosome + number”, whereas a QTL affecting multiple traits was uniformly named as “q + DT (drought tolerance) + chromosome + number” [69].

### 4.4. Transcriptome Sequencing and Data Analysis

Three soybean accessions were selected from the drought-tolerant germplasm for transcriptome sequencing analysis. These accessions were Kefeng No. 1, P556 (from the Yangtze-Huai soybean breeding germplasm population developed by Nanjing Agricultural University [70]), and Zhongpin 16J025; they were renamed Drought-Tolerant 1, 2, and 3 (abbreviated as DT1–DT3), respectively. For each accession, a pure water control group (CK) and a 15% PEG-6000 drought treatment group were established, each with three biological replicates. Radicle tissues were collected 24 h after treatment for transcriptome sequencing, aiming to identify key genes and metabolic pathways involved in the drought stress response of these tolerant accessions. Samples were immediately snap-frozen in liquid nitrogen upon collection and stored at −80 °C in an ultra-low temperature freezer.

Total RNA was extracted using a Plant RNA Extraction Kit (TianGen, Beijing, China) following the manufacturer’s instructions. A total of 18 qualified RNA samples were obtained, and cDNA libraries were constructed using the NEBNext^®^ Ultra™ RNA Library Prep Kit for Illumina (New England Biolabs, Ipswich, MA, USA). Library quality was assessed by initial quantification using a Qubit 2.0 fluorometer, insert size verification using an Agilent 2100 Bioanalyzer, and precise quantification of effective concentration via RT-qPCR, after which high-throughput sequencing was performed on the Illumina platform. Library construction and sequencing were entrusted to Wuhan MetWare Biotechnology Co., Ltd. (Wuhan, China).

Raw sequencing data were subjected to quality control filtering following previously established methods, with adapters and low-quality sequences removed to obtain high-quality clean reads. The clean reads were aligned to the soybean reference genome Williams 82 (Wm82.a2.v1) using HISAT2 v2.1, and read counts for each gene were calculated using FeatureCounts v1.6.2 [71,72]. Gene expression levels were quantified as FPKM (fragments per kilobase of transcript per million fragments mapped) values, which normalize for sequencing depth and transcript length [73]. Expression profiles of the three accessions under PEG treatment versus control conditions were compared, and genes with |log_2_FC| ≥ 1 and Padj < 0.05 that were significantly differentially expressed in all three accessions were identified as differentially expressed genes (DEGs). The resulting DEGs were subjected to GO functional annotation and KEGG pathway enrichment analysis to elucidate the core biological functions and major metabolic pathways underlying the soybean radicle response to drought stress.

### 4.5. Discovery of Candidate Genes Within Key Loci

In this study, potential candidate genes for drought tolerance at the germination stage of soybean were predicted by integrating GWAS and transcriptome sequencing results. This process mainly relied on functional annotation of genes within major loci, their expression profiles under different treatments, and enrichment analysis. Gene functional annotations (Wm82.a2 version) were downloaded from the SoyBase database (SoyBase; http://www.soybase.org). Gene expression data were derived from transcriptome sequencing of three soybean accessions under two treatment conditions. Unlike the enrichment analysis of differentially expressed genes, the criterion of |log_2_ fold change| ≥ 1 may be overly stringent in the context of candidate gene mining. Therefore, with reference to previous studies, genes exhibiting |log_2_ fold change| ≥ 0.5 across all three accessions were also considered potential targets and included in subsequent screening [74,75].

## 5. Conclusions

In this study, a panel of 270 soybean accessions was employed for phenotypic evaluation and genome-wide association study (GWAS) of five drought tolerance indices at the germination stage. Integrating results across all indices and both models resulted in 97 important QTNs distributed across 14 genomic intervals, considered key loci. Notably, qDT-4-2 and qDT-9-1 simultaneously regulated all five indices and were the most important loci. Transcriptome analysis under PEG versus CK conditions revealed 2109 genes commonly expressed in three tolerant accessions. GO and KEGG analyses of these DEGs showed significant enrichment of pathways related to cell wall metabolism, osmotic regulation, and antioxidant defense, which are associated with drought stress responses. Integration of GWAS and RNA-seq data predicted 12 candidate genes within these loci, including those encoding LEA proteins, trehalose-6-phosphate synthase, aquaporin-like superfamily protein, dehydroascorbate reductase, and others involved in drought tolerance-related biological processes. These findings provide insights into the genetic network of drought tolerance during soybean germination and offer useful references for marker-assisted breeding and functional gene discovery.

## Figures and Tables

**Table 1:** Descriptive statistics for five germination-related traits of 270 soybean accessions under CK and 15% PEG-6000 treatments.

Trait ^a^	Mean	SD ^b^	Max	Min	CV ^c^/%
GE-CK/%	49.08	17.83	91.67	10.00	36.34
GR-CK/%	58.82	18.50	96.67	15.00	31.45
RL-CK/cm	5.60	2.76	19.41	0.84	49.19
RFW-CK/mg	124.39	61.40	306.20	14.53	49.36
RDW-CK/mg	11.84	7.05	68.47	1.27	59.55
GE-PEG/%	19.91	12.87	75.00	0.00	64.63
GR-PEG/%	33.55	17.58	80.00	5.00	52.38
RL-PEG/cm	2.59	1.35	11.59	0.25	51.98
RFW-PEG/mg	39.83	18.97	105.33	1.87	47.62
RDW-PEG/mg	6.12	2.98	16.96	0.67	48.67
RGE/%	41.98	20.30	100.00	0.00	48.37
RGR/%	55.76	17.95	100.00	6.16	32.20
RRL/%	53.69	17.89	111.86	7.47	33.32
RRFW/%	42.15	22.58	131.13	1.15	53.57
RRDW/%	57.01	28.58	180.87	2.06	50.13

^a^ CK = ddH2O; ^b^ SD = standard deviation; ^c^ CV = coefficient of variation.

**Table 2:** Summary information of 14 QTLs identified by GWAS.

QTL	Traits	Physical Interval (Mb)	Number of QTNs ^a^	−log_10_*P*	Effect/%	Peak QTN	Reported QTLs/QTNs ^b^
qRGE-1-1	RGE	0.52–0.96	1	4.22	−6.17	Gm01_959343	
qDT-4-1	RRL; RRFW	9.62–9.72	1	3.04	−5.29	Gm04_9618726	qGR4 [11]; Canopy wilt 2-2 [25]
qRRL-4-1	RRL	44.02–44.12	1	4.15	2.41	Gm04_44069560	
qDT-4-2	RGE; RGR; RRL; RRFW; RRDW	44.38–45.42	81	6.55	−11.81	Gm04_45308713	Root morphology 1-3 [26]
qRRL-5-1	RRL	5.21–5.32	1	4.12	4.00	Gm05_5255044	Canopy wilt 2-3 [25]; Drought tolerance 6-2 [27]
qRGR-6-1	RGR	2.85–3.31	1	4.28	−4.64	Gm06_2845683	
qRRL-7-1	RRL	6.45–6.49	1	4.18	3.30	Gm07_6486786	
qRRL-8-1	RRL	43.14–43.44	1	4.86	2.36	Gm08_43146052	
qDT-9-1	RGE; RGR; RRL; RRFW; RRDW	21.54–23.54	2	3.16~8.52	5.09~10.94	Gm09_23064902	
qDT-13-1	RGE; RRFW	40.88–41.01	1	3.37	−10.61~−10.10	Gm13_41011283	qRGP-13-1 [14]
qRGE-15-1	RGE	12.72–12.89	1	3.61~4.35	7.38	Gm15_12789995	Gm15_11950665 [14]
qDT-16-1	RRFW RRDW	4.00–4.12	1 1	3.08	5.73	Gm16_4120259	qWSL16 [11]
qRGE-18-1	RGE	3.27–3.66	1	3.79	−2.50	Gm18_3617601	Root density, lateral 1-1 [28]
qDT-20-1	RRFW RRDW	44.11–44.74	2	7.70	5.08	Gm20_44327627	Root morphology 1-5 [26]

^a^ Number of significant QTNs contained within the QTL. ^b^ Drought-related QTLs/QTNs previously reported in adjacent or overlapping intervals.

**Table 3:** Predicted candidate genes within the key loci.

QTL	Candidate Gene	Arabidopsis Gene	Annotation Description	Biological Processes ^a^
qRGE-1-1	*Glyma.01G007300*	*AT2G19860.1*	Hexokinase	Carbohydrate metabolic process; Glycolysis; Hexokinase-dependent signaling
qDT-4-2	*Glyma.04G180900*	*AT1G23870.1*	Trehalose-6-phosphate synthase	Fructose stimulus; Sucrose stimulus; Trehalose biosynthetic process
qRGR-6-1	*Glyma.06G039000*	*AT1G47380.1*	Protein Phosphatase 2C	
qRRL-7-1	*Glyma.07G071000*	*AT1G63650.* *1*	Transcription factor egl1-related	
qRRL-8-1	*Glyma.08G313500*	*AT5G* *62* *310.1*	Transcription factor jumonji (jmjC) domain-containing protein	
qDT-9-1	*Glyma.09G112100*	*AT5G06760.1*	Seed maturation protein, late embryogenesis abundant (lea) group 1	Response to osmotic stress; Response to water deprivation
qDT-13-1	*Glyma.13G314600*	*AT2G27300.1*	NAC transcription factor gene	Response to salt stress; Seed germination; Seed dormancy process
qRGE-15-1	*Glyma.15G154700*	*AT4G20260.3*	Plasma membrane-associated cation-binding protein 1	Response to calcium ion; Response to osmotic stress; Response to salt stress; Response to water deprivation
qDT-16-1	*Glyma.16G043* *8* *00*	*AT5G18290.2*	Aquaporin-like superfamily protein	Transporter activity; Water channel activity
qRGE-18-1	*Glyma.18G040100*	*AT5G16710.1*	DHAR3, dehydroascorbate reductase	
qDT-20-1	*Glyma.20G210400*	*AT4G14550.1*	IAA61, indole-3-acetic acid responsive gene 61	Lateral root development; Lateral root morphogenesis; Auxin stimulus; Brassinosteroid stimulus
	*Glyma.20G210800*	*AT4G14580.1*	Serine/threonine kinase	Cytokinin biosynthetic process; Protein phosphorylation; Signal transduction

^a^ Candidate gene may be involved in biological processes related to drought tolerance.

## Data Availability

All data generated during this study are included in this published article and its Supplementary Materials. The data presented in this study are available from the corresponding author upon reasonable request.

## Supplementary Materials

The following supporting information can be downloaded at: https://www.mdpi.com/article/10.3390/plants15193036/s1, Table S1: GWAS results of five drought tolerance indices identified by the single-locus model; Table S2: GWAS results of five drought tolerance indices identified by the multi-loci models; Table S3: Summary of transcriptome sequencing data for 18 soybean samples; Table S4: Information on differentially expressed genes (DEGs); Table S5: Results of GO enrichment analysis for all 2109 differentially expressed genes (DEGs); Table S6: Results of KEGG enrichment analysis for all 2109 DEGs; Table S7: Summary of GO enrichment analysis for 430 commonly up-regulated DEGs; Table S8: Summary of GO enrichment analysis for 1679 commonly down-regulated DEGs; Table S9: Expression and annotation information of potential candidate genes within important QTLs; Figure S1: Results of LD block partitioning around important QTNs; Figure S2: Results of principal component analysis (PCA) and inter-sample correlation analysis of transcriptome data for 18 soybean samples; Figure S3: GO enrichment results for commonly upregulated and commonly downregulated DEGs.
