# Supplementary material for: Integrative GWAS and Transcriptome Analysis Reveal Genetic Architecture and Candidate Genes for Drought Tolerance During Soybean Germination
Source: Plants (Basel). 2026 Oct 4;15(19):3036. doi: 10.3390/plants15193036 (PMC13666943; doi:10.3390/plants15193036)
Supplement: Supplementary file 1 [file plants-15-03036-s001.zip › Supplementary Figures.pdf]

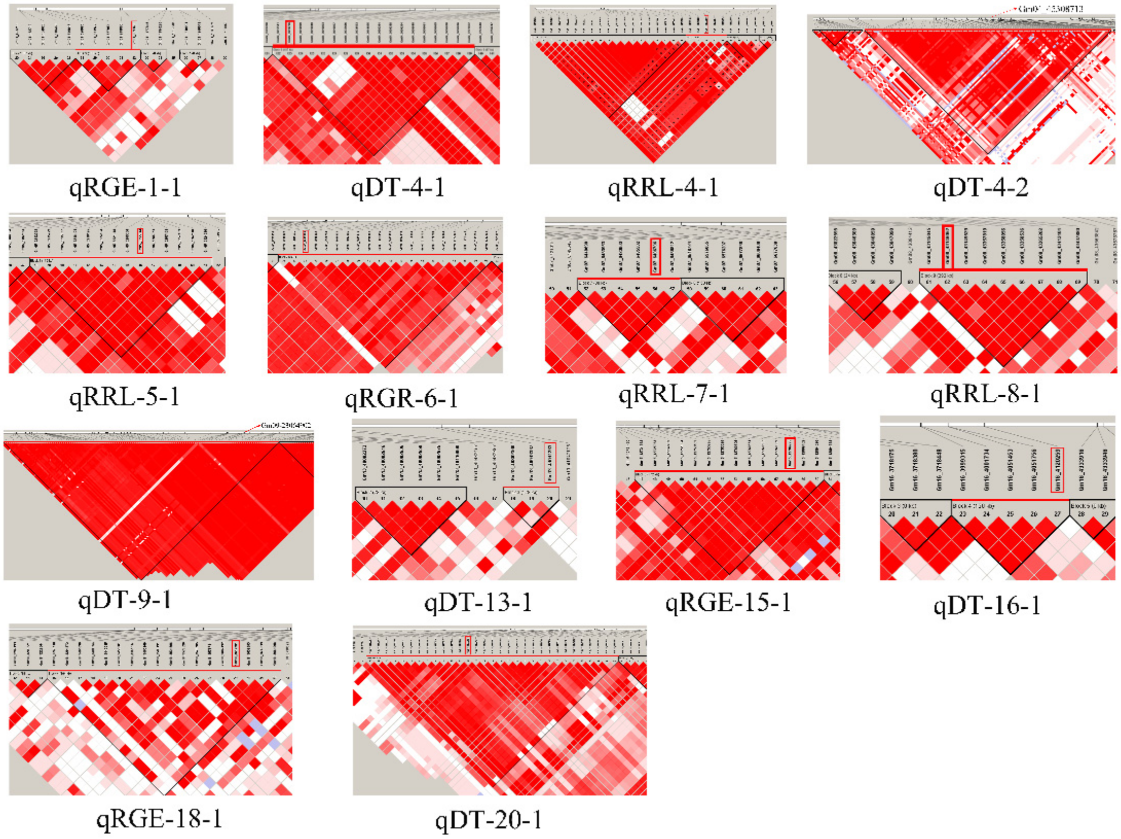

Figure S1. Results of LD block partitioning around important QTNs. Each subplot displays the linkage disequilibrium (LD) among markers within the 14 LD blocks.

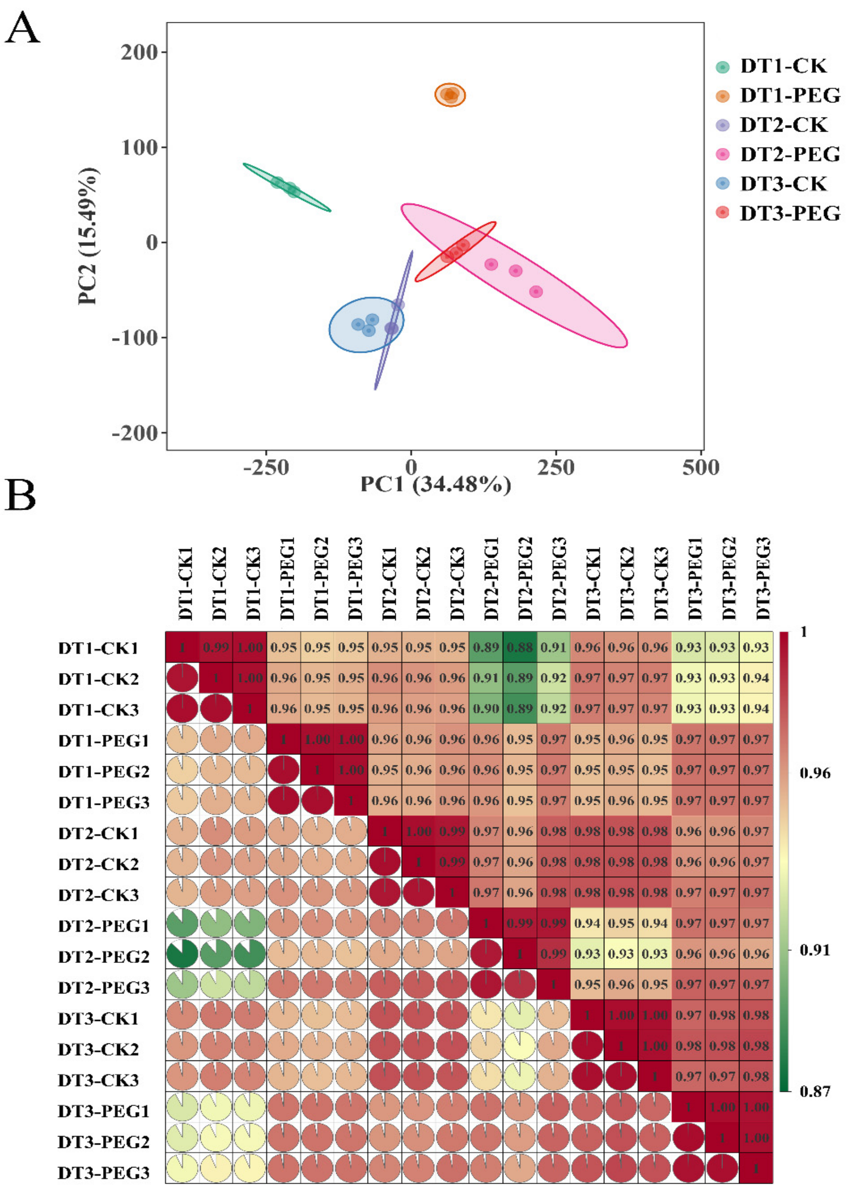

Figure S2. Results of principal component analysis (PCA) and inter-sample correlation analysis of transcriptome data for 18 soybean samples. (A) Principal component analysis (PCA) based on transcriptome expression profiles. (B) Pearson correlation heatmap among samples.

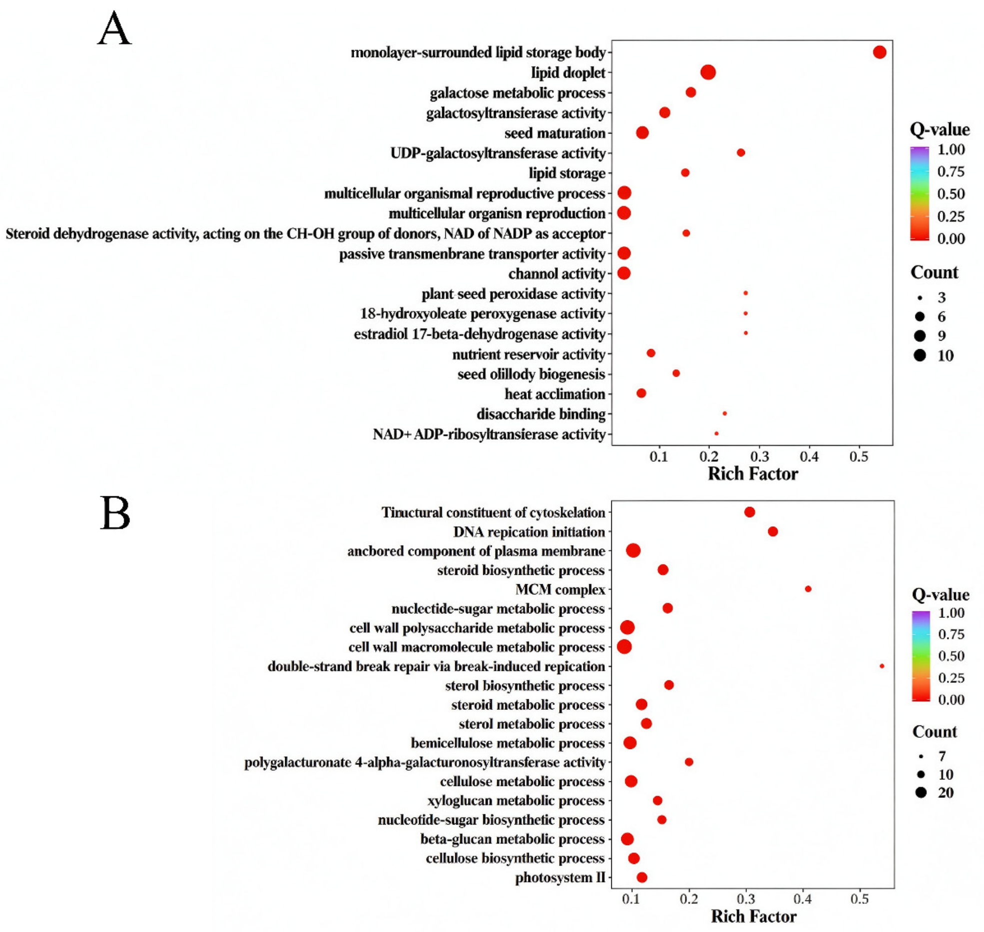

Figure S3. GO enrichment results for commonly upregulated and commonly downregulated DEGs. (A) Commonly upregulated DEGs. (B) Commonly downregulated DEGs.
